# Influence of a vertical subject on research in biomedicine and activities of The Cochrane Collaboration branch on medical students' knowledge and attitudes toward evidence-based medicine

**DOI:** 10.3325/cmj.2012.53.367

**Published:** 2012-08

**Authors:** Karolina Balajić, Vesna Barac-Latas, Ines Drenjančević, Marko Ostojić, Damir Fabijanić, Livia Puljak

**Affiliations:** 1 University of Split School of Medicine, Split, Croatia; 2 University of Rijeka School of Medicine, Rijeka, Croatia; 3 University of J. J. Strossmayer School of Medicine, Osijek, Croatia; 4 University of Mostar School of Medicine, Mostar, Bosnia and Herzegovina

## Abstract

**Aim:**

To investigate whether the introduction of a vertical subject on research in biomedicine and founding of The Cochrane Collaboration branch at the University of Split School of Medicine influenced students’ knowledge and attitudes toward evidence-based medicine (EBM), including the use of research literature.

**Methods:**

We used a 26-item questionnaire on EBM knowledge and attitudes to survey 1232 medical students of all study years in 3 medical schools in Croatia (Split, Rijeka, Osijek) and the Croatian-speaking medical school in Mostar (Bosnia and Herzegovina).

**Results:**

Students from the University of Split School of Medicine who had been exposed to the vertical subject on research in biomedicine and activities of The Cochrane Collaboration at the school had better knowledge and more positive attitudes toward EBM. In general, students rarely searched for evidence; 28% of students searched for evidence more than once a month and 96% of students used only textbooks in Croatian and teachers’ handouts, even though 74% of students agreed that articles from scholarly journals were an important supplement for textbooks.

**Conclusion:**

Building up an environment that fosters EBM may be beneficial for students’ knowledge and attitudes toward EBM. Teachers should encourage and require using evidence during all the courses in medical school.

The term “evidence-based medicine” (EBM) first appeared in the literature in 1992 (1). According to the 1996 definition by David Sackett, “evidence-based medicine is the conscientious, explicit, and judicious use of current best evidence in making decisions about the care of individual patients“ (2). The concept of EBM took a few years to take hold but now it is accepted globally and the use of this term in literature shows a linear increase (3).

The rise of EBM is visible in its gradual incorporation into medical curricula (4-7). Medical students in Bristol, for example, study EBM as a “vertical theme” throughout the undergraduate degree program (8). Pedagogical dimension is central to the EBM initiative and thus the principles of EBM have become core concepts of undergraduate, postgraduate, and continuing medical education (9). The effectiveness of integrating EBM into medical schools’ curriculum in increasing students’ knowledge about EBM has been demonstrated by multiple reports; described interventions range from longitudinal interventions (5) to one-time courses (10).

In 1995, the School of Medicine in Zagreb introduced the course Principles of Research in Medicine into the second year of its medical curriculum (11). Other medical schools in Croatia followed suit. The principles of EBM were the basics of this course. A follow-up study on students’ attitudes toward and knowledge about science showed that attending the course was related to a positive attitude toward science (12). In 2010, the University of Split School of Medicine took another step in teaching research methodology by introducing a vertically and horizontally integrated course on Research in Biomedicine and Health, in an effort to integrate EBM principles into the entire 6-year medical curriculum. This intervention was facilitated by the founding of the Croatian branch of The Cochrane Collaboration at the School in 2008 (13,14).

Both of these activities may have a beneficial effect on medical students’ knowledge and attitudes toward EBM. Therefore, it is essential that a needs assessment exercise is undertaken, particularly because EBM is not uniformly taught in undergraduate education. Such an exercise can provide evidence critical for the development and tailoring of EBM curricula, thus improving the effectiveness of teaching (15). We hypothesized that medical students at the University of Split School of Medicine would have better knowledge and more positive attitudes toward EBM than other Croatian-speaking medical schools, where principles of research methodology are still taught only at the second year. To test this hypothesis, we compared the knowledge and attitudes toward EBM between medical students in Split and three other medical schools. We also assessed students’ exposure to research literature.

## Methods

### Study participants

The study included 1232 medical students of all six years at four Croatian-language medical schools similar in size: the University of Split School of Medicine, University of Rijeka School of Medicine, School of Medicine at the J. J. Strossmayer University of Osijek, all from Croatia, and the University of Mostar School of Medicine in Bosnia and Herzegovina. The study was approved by ethics committees of all four medical schools.

The study was conducted at the end of the academic year 2010/2011. The participants self-administered the questionnaire upon arrival to the teaching session. Student Administration Offices provided the total number of students in each school for response rates calculation.

### Survey

A 26-item questionnaire, based on a validated questionnaire of Hadley et al (15), gathered three types of information – general information and information on knowledge and attitudes. The general information part included information on age, sex, study year, students’ choice of future career, and average grade. Students’ knowledge was assessed by multiple-choice questions about EBM definition, hierarchy of evidence, steps in creating evidence, and importance of EBM. Statements assessing knowledge ranged from 1, indicating insufficient knowledge to 5, indicating excellent knowledge. Statements assessing attitudes toward EBM on a 5-point Likert scale ranged from 1 – strongly disagree to 5 – strongly agree. Furthermore, there were questions about using research studies during medical school, literature used for studying, courses where students had heard about EBM, and students’ perceptions of teachers’ use of EBM.

### Statistical analysis

Descriptive statistical analyses were performed using Microsoft Excel 2007 (Microsoft Corp, Redmond, WA, USA). The data were expressed as frequencies, percentages, medians, and ranges where appropriate. For each attitude question, the five-point Likert scale was collapsed to positive (agree), neutral, and negative (disagree) attitudes. Associations between students’ grades, EBM knowledge, and frequency of using scholarly journals were analyzed using Person correlation and GraphPad Prism (GraphPad software Inc, San Diego, CA, USA). Differences in binary variables were calculated with χ^2^ test. Differences in Likert scale results were calculated with Kruskal-Wallis test, followed by post-hoc Dunn test for differences between each group of data. Statistical significance level was set at *P* < 0.05.

## Results

### Participants

The study included 1232 students, 374 (30%) men and 858 (70%) women. The greatest number of participants was from the medical school in Rijeka (N = 502), followed by Split (N = 286), Osijek (N = 259), and Mostar (N = 185). The number of students per study year ranged from 248 first-year students to 173 sixth-year students. The overall response rate was 63% (1232 respondents out of 1979 registered students). The response rate in Rijeka was 68%, in Split 64%, in Osijek 53%, and in Mostar 59%.

### Knowledge and attitudes toward EBM

More than half of students (n = 778, 64%) responded that they had heard about EBM. The number of students who had heard about EBM increased with each year of study: from 47% of first-year students to 91% of last-year students. The greatest number of students who had heard about EBM were from Split (Table 1), significantly more than from Rijeka (*P* < 0.001), Osijek (*P* < 0.001), and Mostar (*P* < 0.001). The greatest number of students who had heard about EBM already during the first two preclinical study years was also from Split. The number of students who did not respond to the question about the study year when they had heard about EBM was significantly lower in Split than in other cities (Table 1).

**Table 1:** The number of students who heard about evidence-based medicine (EBM)

	No. (%) of students who		
City	had heard about EBM	had heard about EBM during the first and second year	did not reply
Split	269 (94)	238 (83)	80 (43)
Rijeka	229 (46)*	330 (34)*	255 (51)*
Osijek	157 (61)*	132 (49)*	30 (12)*
Mostar	123 (66)*	97 (52)*	116 (45)*

*Significant difference compared with Medical School in Split (*P* < 0.05).

A total of 451 (46%) students recognized the correct EBM definition, 211 (20%) answered correctly to the question about the top-level evidence in the hierarchy of medicine, and 373 (35%) answered correctly to the question about the lowest-level evidence. Students from Split showed significantly better knowledge about the hierarchy of evidence (Table 2). The number of students who answered correctly to all three questions about EBM knowledge was significantly higher in Split than in other cities (Table 2). Compared to Split, students from other cities had more missing responses to the three questions (Table 2).

**Table 2:** Correct answers about evidence-based medicine (EBM) definition and hierarchy of evidence

	No. (%) of students who				
	gave correct answers to the question on				responded to all 3 questions
City	EBM definition	top-level evidence	lowest-level evidence	all 3 questions	
Split	113 (41)	117 (41)	145 (52)	23 (8)	268 (94)
Rijeka	201 (52)*	40 (9)*	129 (29)*	4 (1)*	379 (75)*
Osijek	89 (48)	27 (14)*	55 (28)*	6 (2)*	215 (83)*
Mostar	48 (35)	27 (18)*	44 (31)*	2 (1)*	133 (72)*

*Significant difference compared with Medical School in Split (*P* < 0.05).

Students were not confident about assessing the study design, generalizability, and overall value of a study, or evaluating bias, sample size, and statistical tests (Table 3). Students from Split rated their knowledge in five out of six subjects better than those from Rijeka, Osijek, and Mostar (Table 3). The results on the knowledge scale showed very high consistency (Cronbach α of 1.0).

**Table 3:** Medical students’ self-perceived knowledge about evidence-based medicine (EBM)*

Statements	Split	Rijeka	Osijek	Mostar
Assessing study design	2.01 ± 1.08	1.54 ± 0.88	1.51 ± 0.80^†^	1.76 ± 1.76^†^
Evaluating bias	1.95 ± 1.07	1.59 ± 0.89^†^	1.55 ± 0,81^†^	1.81 ± 1.02
Evaluating sample size	2.13 ± 1.14	1.83 ± 1.00^†^	1.81 ± 1.02^†^	2.16 ± 1.19
Assessing generalizability	2.04 ± 1.11	1.81 ± 1.08^†^	1.75 ± 0.98^†^	1.98 ± 1.06
Evaluating statistical tests	2.00 ± 1.13	1.80 ± 1.03	1.73 ± 0.96	1.97 ± 1.09
Assessing overall value	2.18 ± 1.18	1.86 ± 1.06^†^	1.83 ± 1.02^†^	1.97 ± 1.08

*Responses were measured with grades ranging from 1 = insufficient to 5 = excellent. Data are expressed as mean and standard deviation.

†Significant difference compared with Medical School in Split (*P* < 0.05).

Attitudes toward EBM were analyzed only for students who indicated that they had heard about EBM. More students from Split than from other cities agreed that EBM was useful and essential for clinical practice (Table 4). Students from Split indicated a need to obtain more knowledge on EBM. The results on attitudes were highly consistent (Cronbach α of 0.8).

**Table 4:** Attitudes related to evidence-based medicine (EBM) of students who indicated that they had heard about EBM*

Statements	Split	Rijeka	Osijek	Mostar
EBM is useful for clinical practice	1.96 ± 1.00	1.57 ± 0.86^†^	1.52 ± 0.82^†^	1.69 ± 0.73^†^
EBM is a passing fashion	3.57 ± 1.24	4.05 ± 1.05^†^	4.08 ± 1.06^†^	3.82 ± 1.18
EBM is essential for clinical practice	2.23 ± 1.04	1.78 ± 0.94^†^	1.89 ± 0.90^†^	2.06 ± 0.93
I need more knowledge about EBM	2.37 ± 1.24	1.63 ± 0.87^†^	1.78 ± 0.94^†^	1.97 ± 0.99^†^
Systematic reviews are necessary for clinical practice	2.54 ± 1.14	2.29 ± 0.91	2.25 ± 0.91	2.50 ± 1.07
We learn enough about EBM during medical school	2.68 ± 1.34	3.82 ± 1.15^†^	3.19 ± 1.05^†^	3.08 ± 1.08^†^
An expert opinion is more important than evidence from literature	2.41 ± 1.14	2.66 ± 0.98^†^	2.55 ± 0.94	2.50 ± 1.09
Patients’ wishes are more important than evidence from literature	3.05 ± 1.33	3.33 ± 1.15^†^	3.26 ± 1.18	3.05 ± 1.20

*Responses were measured on a 5-point Likert scale ranging from 1 = completely disagree to 5 = completely agree. Data are expressed as mean and standard deviation.

†Significant difference compared with Medical School in Split (*P* < 0.05).

### Exposure to scientific literature

The main sources of research information were textbooks (N = 489, 44%) and the internet (N = 485, 43%), followed by research databases (N = 219, 20%) and/or scholarly journals (N = 189, 16%).

Students rarely searched for evidence to supplement their standard recommended course literature. Most searched for evidence “fewer than once a month” (N = 482, 42%) or “never” (N = 349; 30%). Out of the third of students who searched for evidence more than once a month, 97 (9%) did it more than once a week.

Students most frequently indicated that they had heard about EBM for the first time in the courses Principles of Research in Medicine (N = 211, 33%), Introduction to Medicine (N = 86, 14%), Physiology/Pathophysiology (N = 82, 13%), and Biology (N = 58, 9%). They mostly used scholarly journals for basic science courses (N = 735, 68%), the top five being Principles of Research in Medicine (N = 265, 35%), Biology (N = 118, 16%), Physiology (N = 58, 8%), Biochemistry (N = 45, 6%), and Internal Medicine (N = 45, 6%), while all other courses were mentioned by less than 5% of students.

The scholarly journals were most frequently accessed via bibliographical databases (N = 470, 45%), medical school library (N = 390, 37%), or the internet (N = 149, 14%), while only a few students borrowed journals from teachers (N = 8, 1%) or purchased the articles (N = 6, 1%). Half of the students (n = 633, 55%) indicated that categorization (impact factor, indexing) of the journal was not important for their choice of journal.

Most of the students (N = 874, 74%) agreed with the statement that articles from scholarly journals were an important supplement for textbooks. Journals were considered important because they provided more recent (N = 477, 43%) and more detailed information than textbooks (N = 227, 20%), and described facts in the context of daily clinical practice (N = 156, 14%), while 249 (22%) students considered scholarly journals completely unimportant for medical school studies.

A total of 1039 (96%) students used textbooks and handouts in their native language and only 43 (4%) supplemented these with literature in foreign languages. Half of the students (N = 603, 50%) read scholarly journals very rarely, 334 (28%) only when it was required by the teachers, 179 (15%) never, and 71 (8%) often. Students perceived that teachers “sometimes” used scholarly journals in their teaching (N = 603, 50%), followed by “regularly” (N = 200, 17%) and “never” (N = 71, 6%), while 335 (28%) students responded that they were not able to estimate this.

A large proportion of students (N = 520, 44%) responded that they were not able to estimate whether teachers from basic or clinical sciences used scholarly journals more. Of those who answered this question, twice as many responded that basic science teachers used scholarly journals more than clinical sciences teachers (25% vs 14%).

A total of 794 (68%) students would like to practice medicine after completing their studies, 51 (4%) were interested in public health, 26 (2%) in a research career, and 299 (26%) were still undecided. Students’ average grade was 4.04 ± 0.48. We did not find an association between students’ grades and the response that they had heard about EBM (Pearson r = -0.04, *P* = 0.46); neither between their grades and the frequency of using scholarly journals (Pearson r = -0.07, *P* = 0.22). Data about the exposure to literature are not shown in the tables.

## Discussion

Our study showed that medical students from the University of Split School of Medicine, who were taught research methodology in all study years and were exposed to the activities of The Cochrane Collaboration branch, had better knowledge of and more positive attitudes toward EBM than students from other medical schools, who were taught research methodology only in one study year. Students from other cities more often did not answer the questions about knowledge, indicating that they did not know the answer or were unsure about its accuracy.

One of the strengths of our study is that we surveyed a large sample of medical students from four medical schools and had good response rate considering the diversity of locations. On the other hand, the limitations of the study could be the questionnaire that relied on the students’ self-perceived assessment of knowledge and beliefs, and the survey design that does not allow the determination of causative relationships. Furthermore, we included smaller Croatian-speaking medical schools that are similar in their programs, size, and development of clinical medicine. School of Medicine in Zagreb is the center of tertiary medical care and clinical research, so medical students in Zagreb may have more opportunities to be exposed to EBM, which is a significant advantage for those students.

In a study among junior physicians of various specialties, Hadley et al have shown that clinicians lack methodological competence necessary for practicing EBM (15). Junior physicians supported the principles of EBM, but were confused regarding whether patient choice and expert opinion were more important than research evidence (15). These observations can be inferred from our data as well, as our respondents generally indicated positive attitudes toward EBM, but had insufficient knowledge of EBM skills and were confused regarding the hierarchy of evidence.

The Medical School Objectives Program, developed by the Association of American Medical Colleges, advocates incorporation of EBM principles into the entire undergraduate education (16). A number of research reports indicated a beneficial effect of EBM curricula and training courses in medical schools. Also, medical students’ knowledge and attitudes toward EBM have improved after EBM training seminars (10). Incorporating EBM into clerkship curriculum improved self-perception of medical students in the key areas of critical appraisal skills, such as formulating a clinical question, searching the literature, and evaluating the evidence; it also increased students’ use of journal literature (17). Assigning each medical student an EBM advisor improved their self-reported attitudes toward EBM and skills for using EBM (18).

EBM is predominantly taught in later years of medical schools (10,19,20), probably because of students' greater experience with patient case studies. However, EBM principles can be taught from the first year, for example by teaching search strategies and evidence assessment during preclinical classes and then reviewing strategies for evaluating different types of articles during clinical rotations (21). Srinivasan et al have shown that an early introduction of EBM principles to preclinical medical students is feasible and practical (22). In a study evaluating different EBM teaching methods in the 8th semester of medical school, students unanimously stated that EBM should be taught earlier in their studies (23).

The schools analyzed in this study had had similar structure of the research methodology courses before 2010/2011 academic year, when the vertical course on research in biomedicine was introduced in Split. Since October of 2010, medical students of all years in Split have been required to attend courses in research methodology, which means that all students included in the study were exposed to new curriculum and teaching on research methodology and EBM. Although it may be too early to judge the new curriculum after only one academic year, better results of students from Split may also be explained by their greater exposure to EBM through the activities of The Cochrane Collaboration branch.

The courses on research methodology at the University of Split School of Medicine were adjusted to students’ needs and medical knowledge. First-year students learned about the types of research studies and general principles of conducting research, while sixth-year students learned practical advanced skills useful in the preparation of their undergraduate theses. West et al have shown that longitudinal medical school EBM courses are linked with markedly increased gains in knowledge about EBM and importance of EBM for medical education and clinical practice (5).

Even before the University of Split School of Medicine introduced the vertical subject on research methodology, it had been very progressive in adopting EBM principles – in 2008 the Croatian branch of The Cochrane Collaboration was founded in Split, involving many teachers and students (13,14). Therefore, medical students in Split have been exposed to EBM through formal and informal learning. Probably this is why they indicated they needed more knowledge on EBM, meaning that they understand that EBM is a wide topic requiring further elaboration.

Although EBM has become a golden standard for clinical practice, Croatian physicians showed insufficient EBM knowledge and usage but considered EBM important for clinical practice (24). These findings are consistent with other studies worldwide (25-27). Possible explanations for this discrepancy between attitudes and knowledge were offered by the respondents themselves – lack of time and skills (6). The key to increase medical students’ and health care workers’ knowledge about EBM is to place more emphasis on EBM during undergraduate and postgraduate, as well as continuing medical education.

Despite positive attitudes toward EBM, we found that students insufficiently used scholarly journals and often used only recommended textbooks and handouts for studying. Although students mostly perceived that their teachers used scholarly journals in preparing their lessons, the number of courses for which they used them was very limited. Landry et al found that improved medical students’ attitudes toward and knowledge of the use of medical literature did not increase the usage of the literature in their written assignments (28).

Bradley et al have reported that medical students were more likely to adopt EBM principles in their learning and clinical experience when there was a definite link between classroom teaching of EBM and clinical application (23). Teaching EBM to medical students in an environment where they can directly apply their EBM knowledge and skills in daily practice may encourage them continue to practice according to the EBM principles (6).

High quality health care implies clinical practice consistent with the current best evidence (29). The EBM has thus become an impetus for incorporating critical appraisal of research evidence alongside routine clinical practice. Increasingly, acquisition of EBM knowledge and skills is becoming a core competence to be acquired by all doctors (15). Our findings may stimulate other schools to reform their curricula and teachers of all medical school courses to encourage their students to search for and use evidence. However, one should distinguish between the students’ knowledge and attitudes toward EBM and actual clinical knowledge and practical skills, especially at the end of the medical studies. We recommend further research about the influence of knowledge and attitudes toward EBM on good clinical practice.
